# Noninvasive cardiac index estimation under general anaesthesia: comparison between the VenArt® device and transthoracic echocardiography☆

**DOI:** 10.1016/j.bjao.2025.100516

**Published:** 2025-12-17

**Authors:** Catherine Paschoud, Nicolas Silvestrini, John Daniels, Jérémie Koegel, Stéphanie Mulin, Florence Gonzalez Ennahdi-Elidrissi, Laszlo Vutskits, Nadia Elia, Georges L. Savoldelli

**Affiliations:** 1 Division of Anaesthesiology, Department of Acute Care Medicine, Geneva University Hospitals, Geneva, Switzerland; 2 Department of Anaesthesiology, Pharmacology, Intensive Care and Emergency Medicine, Faculty of Medicine, University of Geneva, Geneva, Switzerland

**Keywords:** cardiac index, cardiac output, echocardiography, Fick principle, general anaesthesia, laparoscopy, transthoracic echocardiography, VenArt device

## Abstract

**Background:**

VenArt® is a novel noninvasive cardiac output monitoring device which provides beat-by-beat Fick principle-based measurements of stroke volume and cardiac output. The study aim was to determine the accuracy of this device by comparing it with cardiac output measurements using transthoracic echocardiography in patients undergoing anaesthesia.

**Methods:**

This prospective observational study included 55 women (ASA physical status classification I–III) undergoing laparoscopic gynaecological procedures. Cardiac output was assessed at five timepoints using the VenArt® device and transthoracic echocardiography. Primary endpoint was the agreement between the two methods regarding cardiac index, evaluated using Bland–Altman analysis to determine bias, precision, mean percentage error, and limits of agreement. Secondary endpoint was the ability of the device to track changes in cardiac index over time compared with echocardiography.

**Results:**

We analysed 273 pairs of cardiac index values from 55 patients. Bland–Altman analysis showed a bias of 0.02 (95% confidence interval [CI] 0–0.05) L min^−1^ m^−2^, with a precision of 0.20 and a mean percentage error of 14.7% (95% CI 13.2–16.2%). Limits of agreement ranged from −0.37 (95% CI −0.41 to −0.33) to 0.41 (95% CI 0.37–0.45) L min^−1^ m^−2^. Trending ability demonstrated good agreement: the four-quadrant plot revealed a concordance rate of 95.88%, and the polar plot showed a mean polar angle of 0.75°, with a standard deviation of 13.4° and radial limits of agreement within plus or minus 30°.

**Conclusions:**

The VenArt® device showed negligible bias and acceptable differences. Trending ability was favourable, with clinically acceptable agreement and high concordance in tracking haemodynamic changes.

**Clinical trial registration:**

ISRCTN92565809.

Monitoring cardiac output (CO), a key determinant of tissue oxygen delivery and arterial blood pressure, provides us with meaningful information to guide haemodynamic management during the perioperative period.^1^ Although pulmonary artery catheterisation, using thermodilution or the Fick principle, is considered the gold standard for CO measurement, its invasiveness limits routine use in clinical practice.^2^^,^^3^ Transthoracic echocardiography (TTE) provides an appealing noninvasive alternative as this method shows acceptable accuracy and precision when compared with invasive CO measurement.^4^^,^^5^ Accordingly, TTE is widely adopted in perioperative care as it has the additional advantages of being safe and usually feasible during anaesthesia.^6^ It does, however, require a trained operator to ensure the precision of the measurements, occupies space in an often constrained environment, and only provides intermittent measurements.

Over the past 15 yr, an increasing number of medical devices have been made available to provide continuous noninvasive CO assessment.^7^ These devices use a wide variety of technologies, often combined with proprietary algorithms, to estimate CO. While they are not interchangeable with each other or with measurements obtained from invasive assessment, the ‘Best Practice Statement’ from the European Society of Intensive Care Medicine recommends their use for haemodynamic monitoring and optimisation, after validation.^8^ Validation studies are of utmost importance before clinical applicability and are aimed to evaluate the accuracy, precision, and trending ability of these devices when compared with a reference method.^9^ Given the good agreement between pulmonary artery catheterisation and TTE for CO measurement, this latter is frequently used in otherwise healthy individuals as a reference method.

VenArt® (Mespere LifeSciences, Waterloo, ON, Canada) is a relatively new device which provides noninvasive CO determination by combining a conventional pulse oximeter probe with a near-infrared spectroscopy (NIRS) sensor placed over the external jugular vein to determine the difference between arterial and venous oxygen saturations. Through a proprietary algorithm, based on the Fick principle, it allows for stroke volume (SV) and CO estimation. While this device is marketed for clinical use, there are so far no publicly available data showing its accuracy when compared with any reference method.

The present study was therefore designed to address this question. By focusing on a cross-sectional study of women undergoing laparoscopic gynaecological procedures, we aimed to determine how cardiac index derived from CO measurements with the VenArt® device compared with cardiac index derived from simultaneous CO measurements by TTE in terms of bias, precision, mean percentage error (MPE) and limits of agreement (LoA). We also evaluated the ability of the VenArt® device to track changes in cardiac index over time, in comparison with TTE.

## Methods

### Ethics

Ethics approval for this study (BASEC 2023-02000) was provided by the Commission Cantonale d’Ethique de la Recherche (CCER), Geneva, Switzerland (Chairperson Dr Olivier Huber) on 13 February 2024. All patients gave written informed consent. The study protocol was registered in the ISRCTN registry (ISRCTN92565809) and conducted in accordance with the Helsinki Declaration, the principles of Good Clinical Practice, the Human Research Act (HRA) and the Human Research Ordinance (HRO).^10^^,^^11^ It is available from the corresponding author upon reasonable request.

### Study design

This prospective, observational, monocentric clinical study compared Venart® cardiac index values with cardiac index derived from TTE measures. Data collection was prospectively planned before performing both measurement methods. The study was conducted by the Division of Anaesthesiology and the Faculty of Medicine of the Geneva University Hospitals (Geneva, Switzerland) between April and October 2024, and the reporting follows the STARD (Standards for Reporting Diagnostic accuracy studies) 2015 recommendations.^12^

### Participants

We included adult women (≥18 yr) scheduled for elective gynaecological laparoscopy expected to last >60 min under general anaesthesia, with an ASA physical status classification of I–III, who were able to read and understand French. The operating schedule was screened for all potentially eligible patients between April and October 2024. All consecutive patients who agreed to participate were recruited provided that the research team was available on the day of surgery.

Exclusion criteria were a BMI >35 kg m^−2^, any condition limiting the use of VenArt® device (according to the user manual, e.g. very restricted neck anatomy, abnormalities in peripheral saturations, known allergy to adhesive products—skin sensor, central vein stenosis, Reynaud's disease, severe chronic obstructive pulmonary disease), any condition limiting the feasibility of TTE (unplanned surgeries [emergency], aortic regurgitation), and laparoscopic procedures using vaginal natural orifice transluminal endoscopic surgery (vNOTES).

### Study measurements

Patients’ characteristics (age [yr], weight [kg], height [cm], and ASA physical status) were collected before surgery. During anaesthesia preparation, the VenArt® neck sensor was placed over the external jugular vein and the pulse oximeter probe attached to a finger; neither device required calibration.

Signal integrity was maintained for both the neck sensor and the pulse oximeter finger probe throughout the perioperative period. Sensor displacement was uncommon, including during intubation, Trendelenburg positioning, and pneumoperitoneum. The VenArt® device was applied according to manufacturer’s instructions, which do not require ultrasound localisation of the external jugular vein. It has CE marking and its performance is annually certified by the biomedical engineering department according to institutional policy.

CO measurements from both the VenArt® device and from TTE were obtained at five time points: (1) before induction of general anaesthesia, (2) after induction, (3) before surgical incision, (4) after insertion of the first laparoscopic port and establishment of pneumoperitoneum using carbon dioxide at 20 mm Hg, and (5) at least 10 min after reduction of pneumoperitoneum pressure to 12 mm Hg and with a 20–25° Trendelenburg table tilt.

TTE was performed using a 5 MHz cardiac probe with a standard echocardiography machine (EPIQ 7; Philips, Amsterdam, The Netherlands). Aortic diameter was measured via the parasternal long-axis view and aortic flow was assessed using pulsed Doppler across the aortic valve from the apical five-chamber view.^13^^,^^14^ All echocardiographic measurements were performed by the same certified sonographer (JD) to minimise interoperator variability. All spectral velocity time integral tracings were performed after the intervention to avoid bias with the CO displayed by the Venart® device during anaesthesia, meaning the assessor was blinded to the VenArt® CO values.

As the VenArt® device provides continuous CO monitoring, readings were recorded simultaneously with each corresponding set of TTE spectral traces (three sets of three traces per measure).

A member of the research unit, not otherwise involved in the study, monitored the informed consent forms, inclusion and non-inclusion criteria, and all CO measures for all participants. These monitoring checks were performed on 8 July 2024 and on 20 November 2024.

### Sample size

We determined the sample size according to a power analysis using PASS software (version 22.0.7, NCSS, East Kaysville, UT, USA) based on an expected bias (mean of differences) of 0 and a precision (standard deviation) of 1.18. With a confidence interval (CI) of 0.95, the power fixed at 0.8, and a maximum allowable difference of 0.5, 49 subjects with analysable cardiac index were needed.^15^ To account for 10% attrition, we planned to enrol 55 patients.

### Statistical analysis

To ensure conformity, all cardiac index values were calculated from CO derived from both the VenArt® device and the TTE using the same body surface area formula (Mosteller formula).^16^ Cardiac index was calculated as CO divided by patient’s body surface area. We assessed the agreement between the VenArt® device and TTE measures following a Bland–Altman analysis considering the TTE measures as the reference method.^17^^,^^18^ Accordingly, we computed the bias as the mean difference between cardiac index values (VenArt® – TTE), the precision as the standard deviation (sd) of the bias, and the LoA as the bias plus or minus 1.96×sd. The MPE was calculated as 1.96×sd/mean cardiac index×100 and we considered values <30% as clinically acceptable.^19^ Further, we reported 95% CI of the bias, LoA, and MPE.

Additionally, we considered the recommendations of Montenij and colleagues^20^ regarding potential pitfalls in the Bland–Altman analysis. This involved four steps. (1) Checking whether the bias followed a normal distribution using a graphical approach and a Shapiro–Wilk test. Analysis suggested the contrary (*P*<0.001). Therefore, we used a non-parametric approach by computing LoA as the values outside of which a certain proportion of the observations fell (i.e. 10%, above the 95th percentile and below the 5th percentile) and evaluated whether the Bland–Altman analysis was less conservative than the non-parametric approach.^21^ (2) To determine whether we should apply a correction for paired measurements, we computed auto-correlation and Spearman’s rank correlations between measurements, which proved non-negligible (*r*_s_>0.31). Therefore, we used the correction proposed by Bland and Altman^21^ to assess the impact of repeated measurements on our findings. (3) Regarding the precision of the reference method and its potential impact on the 30% criteria for MPE, we computed combined repeatability (i.e. the maximal variation in repeated experimental and reference measurements that could explain the mean error). For the techniques to be considered interchangeable, their combined repeatability should not exceed the MPE. (4) Finally, we assessed the presence of a proportional bias with a mixed-effects model to estimate the slope of the regression line in the relationship between TTE cardiac index values and cardiac index differences. A significant slope would indicate a proportional bias (i.e. a non-uniform bias over the range of measurements).

For trending analysis, we conducted four-quadrant plot (with associated concordance rate) and polar plot analyses.^22^^,^^23^ A concordance rate >90%, a mean polar angle or angular bias of less than plus or minus 5°, an sd for the polar angle of less than plus or minus 15°, and radial LoA of less than plus or minus 30° indicates reliable trending ability.

Statistical significance was assessed at a two-sided 0.05 level. We performed all analyses using R Statistical Software (version 4.3.1; R Core Team, 2021, R Foundation for Statistical Computing, Vienna, Austria).

## Results

**Fig 1:**
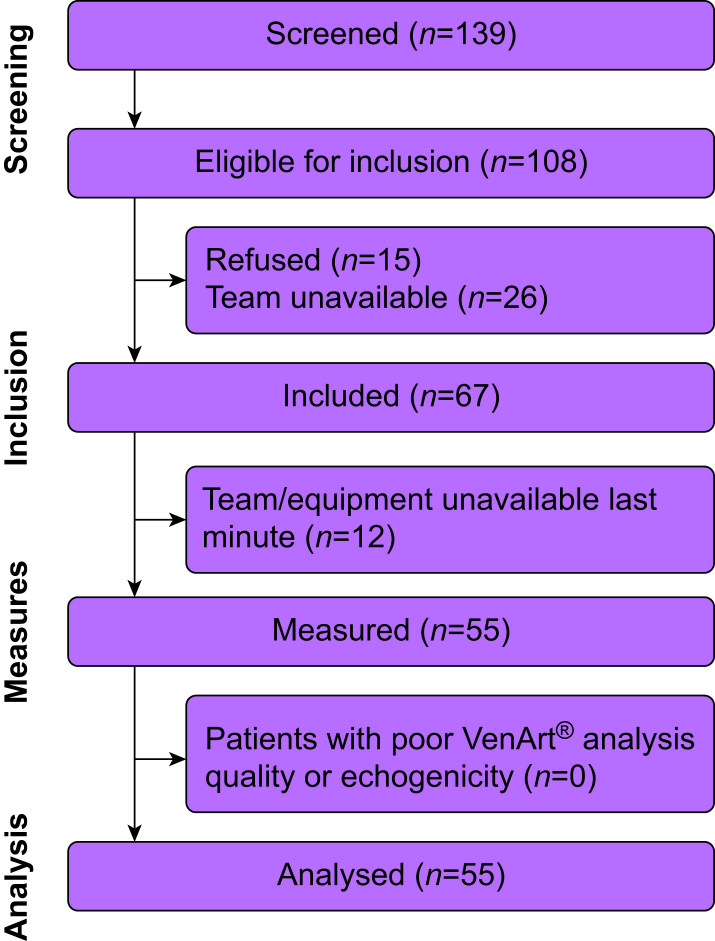
Patient flow chart. Diagram illustrating the flow of patients in the study.

Fifty-five women were included. We obtained five VenArt® device/TTE pairs of CO measurements for each patient; only two measurement sets were lost for analysis, meaning a total of 273 distinct pairs of CO measurements were analysed (Fig. 1). Patients’ characteristics are presented in Table 1. Calculated cardiac index values obtained at each time point are summarised in Table 2. No adverse events occurred.

**Table 1:** Patients’ sociodemographic and clinical characteristics. For continuous variables: mean (standard deviation) (range). For categorical variables: count (%).

	Total (*n*=55)
Age (yr)	44.1 (10.8) (23–77)
Weight (kg)	66.5 (11.4) (44–93)
Height (cm)	163.7 (6.3) (149–176)
BMI (kg m^−2^)	24.9 (4.4) (16.6–33.7)
ASA	
1	8 (14.5)
2	45 (81.8)
3	2 (3.6)

**Table 2:** Calculated cardiac index values obtained using VenArt® and TTE at the five perioperative points. Cardiac index expressed as mean (standard deviation) (95% confidence interval). Units are L min^−1^ m^−2^. TTE, transthoracic echocardiography.

	Time 1 (*n*=55)	Time 2 (*n*=55)	Time 3 (*n*=55)	Time 4 (*n*=54)	Time 5 (*n*=54)
VenArt*®*	2.77 (0.46) (2.64–2.89)	2.39 (0.40) (2.28–2.50)	2.57 (0.43) (2.45–2.68)	2.54 (0.48) (2.41–2.67)	3.05 (0.40) (2.94–3.16)
TTE	2.76 (0.48) (2.63–2.89)	2.38 (0.36) (2.29–2.48)	2.50 (0.42) (2.39–2.61)	2.54 (0.50) (2.40–2.68)	3.02 (0.40) (2.91–3.13)

## Bland–Altman analysis

**Fig 2:**
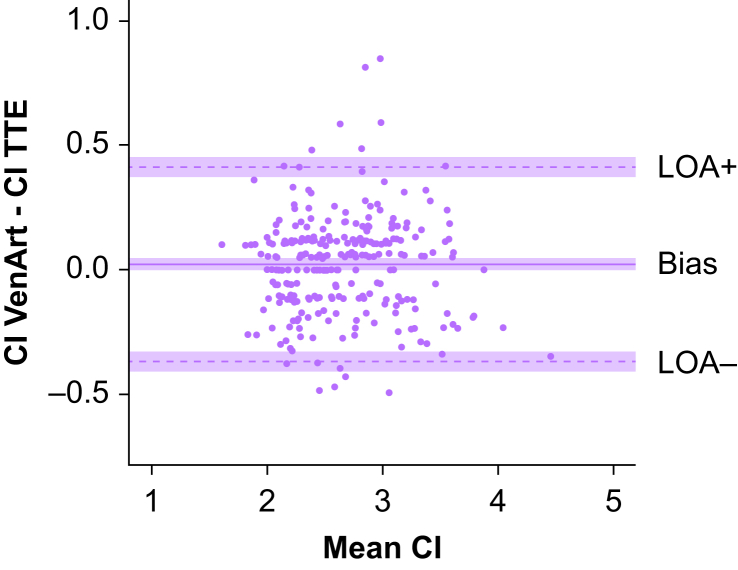
Bland–Altman plot comparing CI values of VenArt® and TTE. Purple areas represent 95% confidence interval for the bias, LoA+ and LoA−. Units are L min^−1^ m^−2^. CI, cardiac index; LoA, limit of agreement; TTE, transthoracic echocardiography.

We conducted a Bland–Altman analysis to assess the agreement between cardiac index derived from the VenArt® device and TTE. The bias was 0.02 (95% CI 0–0.05) L min^−1^ m^−2^ with a precision of 0.20 L min^−1^ m^−2^. The upper LoA was 0.41 (95% CI 0.37–0.45) L min^−1^ m^−2^ and the lower LoA was −0.37 (95% CI −0.41 to −0.33) L min^−1^ m^−2^ (Fig. 2). Most importantly, the MPE was 14.7% (95% CI 13.2–16.2%), which is below the recommended criteria of 30%.

To correctly assess the validity of our method comparison, we applied the recommended checks as proposed by Montenij and colleagues.^20^ The non-parametric approach revealed a LoA+ (95th percentile) of 0.32 L min^−1^ m^−2^ and an LoA− (5th percentile) of −0.29 L min^−1^ m^−2^ (see non-parametric Bland–Altman plot in Supplementary Fig. S1), which were smaller than the LoA computed with the parametric approach.

The LoA+ and LoA− corrected for paired measurements were 0.43 L min^−1^ m^−2^ and −0.39 L min^−1^ m^−2^, respectively (i.e. very close to the non-corrected values).

The MPE was 14.7%, which did not exceed the combined repeatability of TTE (reference method) and VenArt® (test method), calculated at 40.6%.

Finally, the slope of the regression line was close to 0 and not significant (*P*>0.785), which therefore suggested a uniform bias over the range of measurements as presented in an enhanced Bland–Altman plot (see Supplementary Fig. S2).

### Trending ability

**Fig 3:**
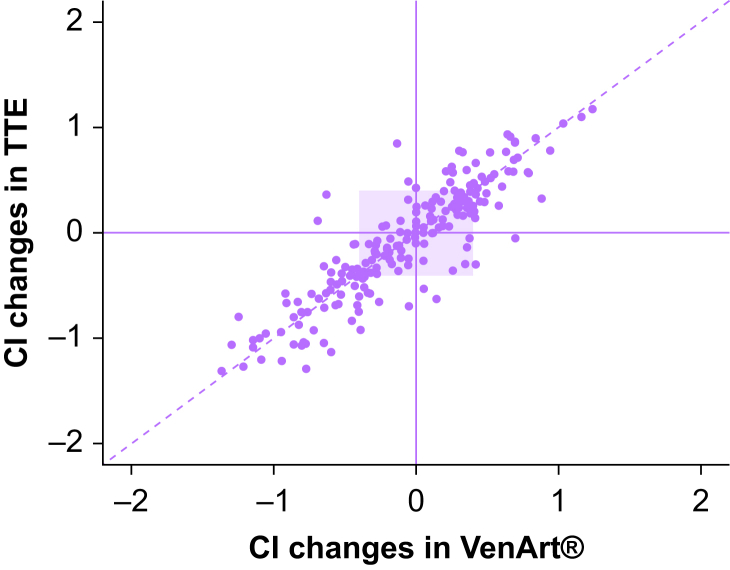
Four-quadrant plot comparing CI changes using TTE and VenArt®. The central purple square area represents the exclusion zone of 15% of the mean cardiac index change. Units are L min^−1^ m^−2^. CI, cardiac index; TTE, transthoracic echocardiography.

**Fig 4:**
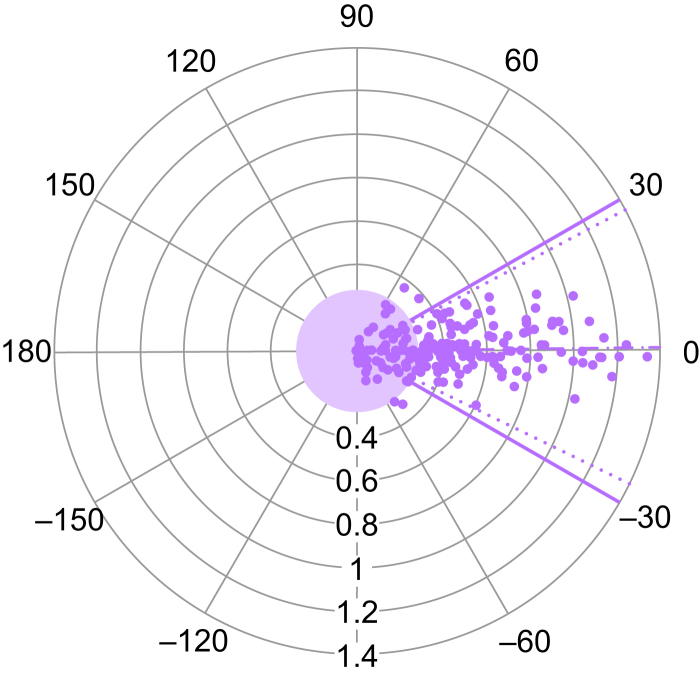
Polar plot comparing CI changes using TTE and VenArt® described as polar coordinates. The central round purple area represents the exclusion zone of 15% of the mean cardiac index change, scaled down by a factor of 1.5. Dashed lines represent mean polar angle and radial LoA. CI, cardiac index; LoA, limit of agreement; TTE, transthoracic echocardiography.

We performed four-quadrant and polar plot analyses to assess the trending ability of the VenArt® device compared with TTE. The four-quadrant plot, incorporating a 15% exclusion zone (0.40 L min^−1^ m^−2^, based on a mean cardiac index of 2.65 L min^−1^ m^−2^) demonstrated a concordance rate of 95.9% (Fig. 3). The polar plot revealed a mean polar angle of 0.75°, a precision (sd) of 13.4°, an upper radial LoA of 27.5°, and a lower radial LoA of −26.0° (Fig. 4), meeting established criteria for acceptable trending performance.^23^

To complement the main analysis, we performed the same agreement and trending assessments using CO values. Bias, precision, LoA, and MPE from the Bland–Altman analysis comparing cardiac index and CO values of the VenArt® device and TTE are summarised in Table 3. The corresponding Bland–Altman plot, four-quadrant plot, and polar plot for CO are provided in Supplementary Figures S3–S5. These analyses produced similar results to those obtained with cardiac index, supporting the consistency of our findings across both metrics.

**Table 3:** Summary of the Bland–Altman analysis comparing VenArt® *vs* TTE for cardiac index and cardiac output values. Values are reported as bias, precision (standard deviation of the bias), limits of agreement and mean percentage error (MPE) along with their 95% confidence interval (CI). Units are L min^−1^ m^−2^ for cardiac index, L min^−1^ for cardiac output and % for MPE. TTE, transthoracic echocardiography.

	Value cardiac index	Value cardiac output
Bias, mean (95% CI)	0.02 (0–0.05)	0.04 (−0.01 to 0.08)
Precision (standard deviation of the bias)	0.20	0.34
Upper limit of agreement (95% CI)	0.41 (0.37–0.45)	0.71 (0.64–0.78)
Lower limit of agreement (95% CI)	−0.37 (−0.41 to −0.33)	−0.64 (−0.71 to −0.56)
Mean percentage error (95% CI)	14.7 (13.2–16.2)	14.6 (13.1–16.2)

## Discussion

Our investigations revealed that the recently marketed VenArt® noninvasive CO monitor showed a clinically acceptable agreement when compared with noninvasive CO measurement performed by TTE. The bias was negligible, the precision was narrow, and the MPE remained well below the commonly accepted 30% threshold, supporting the device’s potential interchangeability with TTE. We also found good correlation in the trending ability of this device, when compared with TTE, to detect changes in CO during the perioperative period. Specifically, the four-quadrant plot showed a concordance rate above 90%, the polar plot revealed a mean polar angle close to zero, an sd for the polar angle of less than plus or minus 15°, and radial LoA within plus or minus 30°, all consistent with the established criteria for acceptable trending performance.^23^ Together, these results support the VenArt® device as a reliable noninvasive method for perioperative CO monitoring in this patient population. These findings indicate that the VenArt® could be used as a simple, noninvasive device for perioperative CO monitoring. Its values closely match those from TTE, without the expertise or time required for the latter. Furthermore, it reliably detects changes in CO perioperatively, making it a useful tool to monitor haemodynamic trends during surgery.

In our study, we chose to analyse and report cardiac index instead of absolute CO because, as suggested by Saugel and colleagues,^24^ cardiac index might be more appropriate to account for individual biometric variability and therefore appropriate decision-making. While both CO and cardiac index are valid parameters, other measures such as SV could also have been considered, as both TTE and the VenArt® device calculate SV from different input variables. In fact, SV is the primary physiological value from which CO and cardiac index are derived, meaning that the comparison between the two devices ultimately rests on their ability to estimate SV accurately. Nevertheless, most method comparison studies report CO, cardiac index, or both, and both metrics generally demonstrate similar results in terms of agreement and trending performance.^24^ This was also confirmed in our dataset as Bland–Altman analysis using CO values showed very similar values for agreement and trending ability to those based on cardiac index.

We found good agreement between the VenArt® device and TTE, with a negligible bias and an MPE of 14.7%, well below the commonly accepted threshold. This level of agreement exceeds that reported in several other studies of other noninvasive CO measurement devices.^19^ For instance, studies comparing TTE with continuous waveform analysis (Nexfin) or ECG-estimated continuous cardiac output (esCCO, Nihon Kohden®, Tokyo, Japan) reported higher biases and MPE exceeding the 30% clinically threshold, along with broader LoA.^25^^,^^26^ This highlights the difficulty to accurately assess CO measurement devices, especially when both the test and reference methods cannot be considered fully accurate. Even though the Bland–Altman analysis is the most widely accepted tool for method comparison analysis, there is still uncertainty on what constitutes clinically acceptable agreement. The 30% MPE threshold, proposed by Critchley and Critchley,^19^ may be too permissive or arbitrary depending on the clinical context. To address these limitations, Montenij and colleagues^20^ recommended checks—accounting for repeated measures, proportional bias, and non-parametric distributions, all of which gave coherent results in our study.

In addition to good agreement between the two methods, we also found strong trending ability in detecting haemodynamic changes, with a concordance rate of 95.9% in the four-quadrant plot and a polar plot meeting the established criteria for acceptable trending ability. Assessing trending ability has crucial clinical relevance, as it reflects how well a device can track changes in CO over time. What matters during the perioperative period is whether a device reliably tracks changes, for example in response to interventions such as fluid challenge, vasopressors administration or changes in ventilation.^27^ These provide the clinician with valuable information to support dynamic decision-making.

Despite the clinical importance of trending ability and the recommendation to obtain four-quadrant plot and polar plot analyses, many validation studies on CO measurements fail to assess them. Overlooking such results might lead to incomplete or inaccurate conclusions regarding whether devices are reliable in real-life perioperative settings. Our results not only showed that the VenArt® device agrees with TTE, but that it accurately tracks perioperative haemodynamic changes over time.

Validation studies to assess the accuracy, precision, and trending ability of CO/cardiac index monitoring devices against a reference method are of the utmost importance before clinical applicability.^9^ However, they are not always performed or published by the manufacturer. Formal validation studies comparing the VenArt® device with a more robust invasive measurement method in clinical settings including emergency surgery or patients with severe co-morbidities, initiated by the manufacturer, would be welcome.

Our study has limitations. We used TTE as a reference method instead of pulmonary artery catheterisation. The latter, although often considered a gold standard for CO/cardiac index measurement, is invasive and carries risks such as arrhythmias, infection, and vascular complications, which, in a low-risk, haemodynamically stable population, would not be ethically or clinically justified. In addition, TTE is widely accepted in perioperative monitoring and provides reliable measurements, making it an appropriate alternative reference method for this type of study.^28^

Second, a limitation of our study is that all TTE measurements were performed by a single sonographer. While this approach ensured consistency and reduced intra-observer variability, it may affect the external reproducibility of our results.

Third, the population consisted exclusively of women undergoing laparoscopic gynaecological procedures. We chose this population because recruitment was straightforward and laparoscopic surgery induces substantial changes in CO over a short period of time. However, this limits the generalisability of our findings to other patient groups, and the clinical relevance of the VenArt® device in sicker, more complex patients—who are likely to benefit most from such monitoring—remains to be demonstrated. Indeed, our results cannot be extrapolated to patients with CO outside the range observed in the present study.

Fourth, we did not evaluate the device in specific pathologies such as obesity (BMI >35 kg m^−2^ being an exclusion criterion), sepsis, arrhythmias, or cardiopathy (only patients with ASA physical status class I–III were included). Indeed, such conditions can lead to poor image quality, inaccurate peripheral measurements or reduced sensitivity in structural heart disease.^7^

Finally, we were unable to evaluate trending ability under extreme haemodynamic conditions or in patients with significant cardiovascular pathology. While these aims were beyond the scope of the study, they certainly warrant further investigation.

### Conclusions

The VenArt® device demonstrated good agreement and trending ability with TTE. These findings suggest that the VenArt® device is reliable for noninvasive cardiac index monitoring during the perioperative period in gynaecological laparoscopic procedures. However, further investigations need to confirm its validity during other surgical procedures, in more complex patients, and under unstable haemodynamic conditions.

## Authors’ contributions

Study conception: CP, JK, LV, NE, GS

Study design: CP, JK, FE, LV, NE, GS

Study coordination: FE, SM

Data acquisition: JD, SM

Interpretation: CP, NS, JD, SM, FE, LV, NE, GS

Statistical analyses: NS

First draft of the manuscript: CP

Critical reviewing of the manuscript: NS, JD, JK, SM, FE, LV, NE, GS

Final approval of the version to be published and agree to be accountable for all aspects of the work: all authors

## Funding

Departmental resources.

## Declarations of interest

The authors declare that they have no conflicts of interest.
