# Supplementary material for: Noninvasive cardiac index estimation under general anaesthesia: comparison between the VenArt® device and transthoracic echocardiography
Source: BJA Open. 2025 Dec 17;17:100516. doi: 10.1016/j.bjao.2025.100516 (PMC12771355; doi:10.1016/j.bjao.2025.100516)
Supplement: Multimedia component 1 [file mmc1.pdf]

Non-invasive cardiac index estimation under general anaesthesia: comparison between the VenArt® device and transthoracic echocardiography.

SUPPLEMENTARY FILES

Supplementary Figure S1: *Nonparametric Bland-Altman Plot comparing CI values of VenArt® and TTE.*

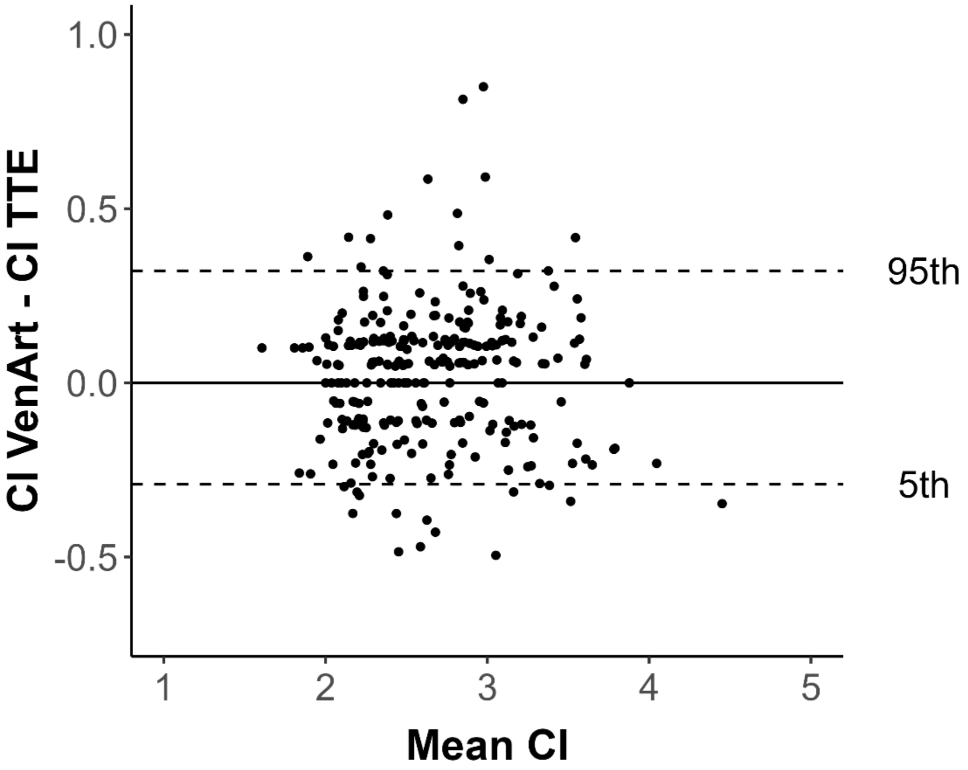

Nonparametric Bland-Altman plot comparing CI values of VenArt® and TTE. Limits of agreement were defined as the 5th and 95th percentiles of the distribution of differences. Units are litre per minute per square metre (l min^{-1} m^{-2}). CI, Cardiac Index. TTE, Transthoracic Echocardiography.

Supplementary Figure S2: *Enhanced Bland-Altman Plot assessing proportional bias between VenArt® and TTE.*

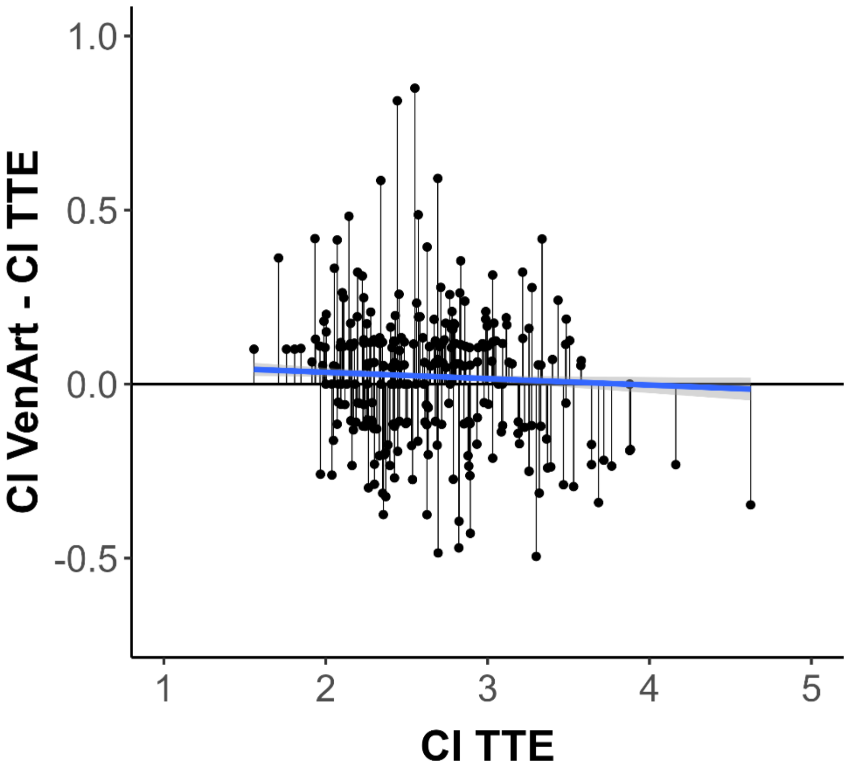

Data points represent the difference (VenArt® – TTE) plotted against their mean. The blue regression line (the grey areas represent 95% CI) is displayed to assess proportional bias; the non-significant slope indicates that the bias is uniform over the measurement range. Units are litre per minute per square metre (l min^{-1} m^{-2}). CI, Cardiac Index. TTE, Transthoracic Echocardiography.

Supplementary Figure S3: *Bland-Altman plot comparing CO values of VenArt® and TTE.*

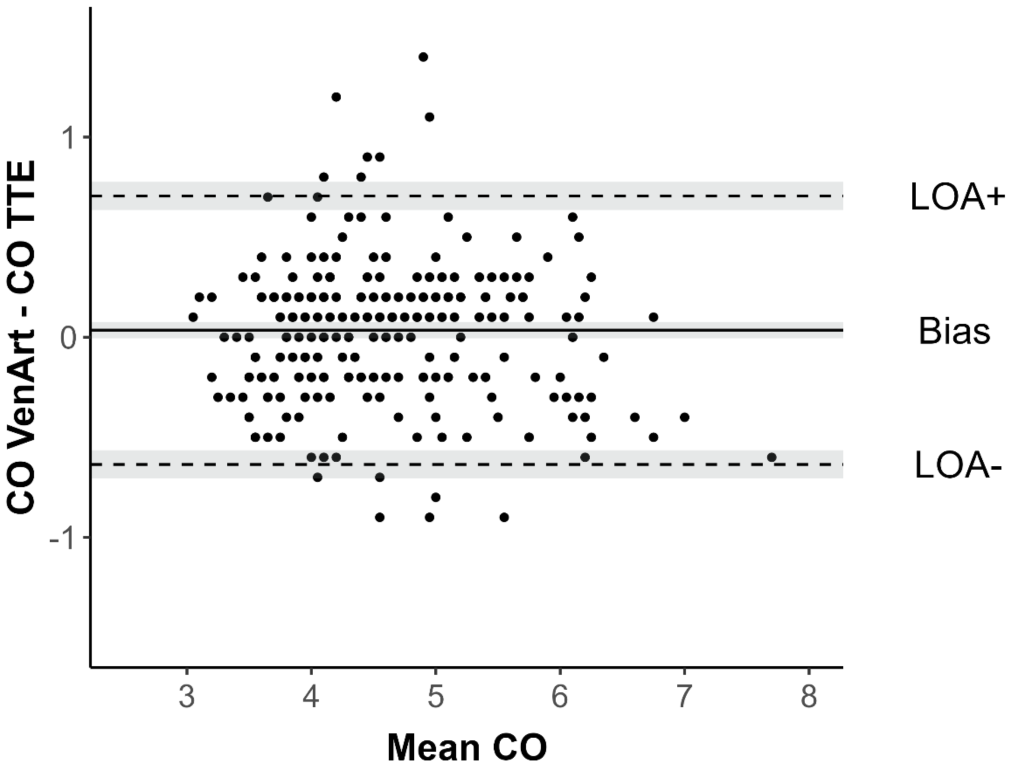

Bland-Altman plot comparing CO values of VenArt® and TTE. Grey areas represent 95% CI for the bias, LoA+ and LoA-. Units are litre per minute (l min^{-1}). CO, Cardiac Output. TTE, Transthoracic Echocardiography. LoA, Limit of Agreement.

Supplementary Figure S4: *Four-quadrant plot comparing CO changes using TTE and VenArt®.*

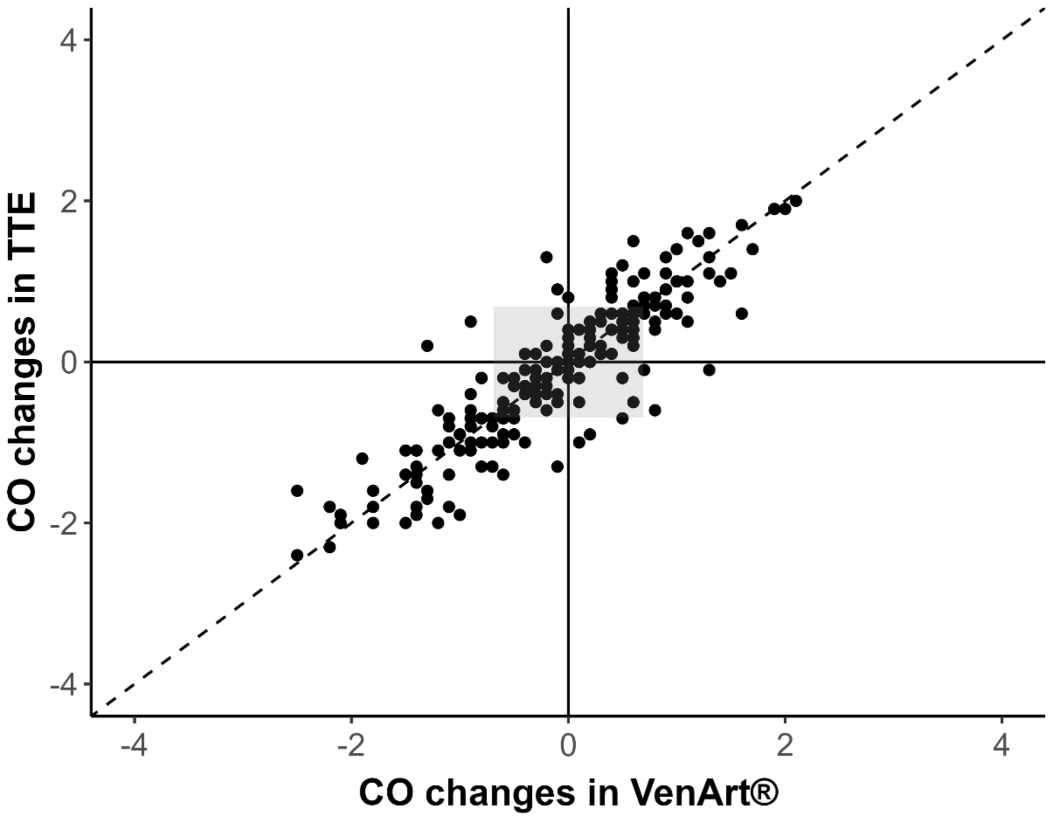

Four-quadrant plot comparing CO changes using TTE and VenArt®. The grey area represents the exclusion zone of 15% of the mean CO change. Units are litre per minute (l min^{-1}). CO, Cardiac Output. TTE, Transthoracic Echocardiography.

Supplementary Figure S5: *Polar plot comparing CO changes using TTE and VenArt® described as polar coordinates.*

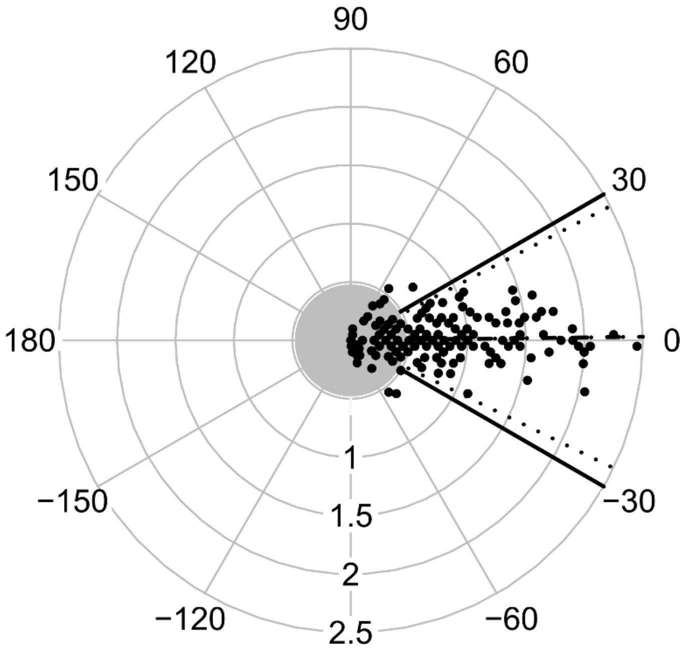

Polar plot comparing CO changes using TTE and VenArt® described as polar coordinates. The grey area represents the exclusion zone of 15% of the mean CO change, scaled down by a factor of 1.5. Dashed lines represent mean polar angle and radial LoA. CO, Cardiac Output. TTE, Transthoracic Echocardiography. LoA, Limit of Agreement.
